# Faller Classification in Older Adults Using Wearable Sensors Based on Turn and Straight-Walking Accelerometer-Based Features

**DOI:** 10.3390/s17061321

**Published:** 2017-06-07

**Authors:** Dylan Drover, Jennifer Howcroft, Jonathan Kofman, Edward D. Lemaire

**Affiliations:** 1 Department of Systems Design Engineering, University of Waterloo, Waterloo, ON N2L 3G1, Canada; djdrover@uwaterloo.ca (D.D.); jirwin02@gmail.com (J.H.); 2 Centre for Rehabilitation Research and Development, Ottawa Hospital Research Institute, Ottawa, ON K1H 8M2, Canada; elemaire@ohri.ca; 3 Faculty of Medicine, University of Ottawa, Ottawa, ON K1H 8M5, Canada

**Keywords:** wearable sensors, machine learning, accelerometer, faller classification, faller prediction, feature selection, elderly, falls, prospective fallers

## Abstract

Faller classification in elderly populations can facilitate preventative care before a fall occurs. A novel wearable-sensor based faller classification method for the elderly was developed using accelerometer-based features from straight walking and turns. Seventy-six older individuals (74.15 ± 7.0 years), categorized as prospective fallers and non-fallers, completed a six-minute walk test with accelerometers attached to their lower legs and pelvis. After segmenting straight and turn sections, cross validation tests were conducted on straight and turn walking features to assess classification performance. The best “classifier model—feature selector” combination used turn data, random forest classifier, and select-5-best feature selector (73.4% accuracy, 60.5% sensitivity, 82.0% specificity, and 0.44 Matthew’s Correlation Coefficient (MCC)). Using only the most frequently occurring features, a feature subset (minimum of anterior-posterior ratio of even/odd harmonics for right shank, standard deviation (SD) of anterior left shank acceleration SD, SD of mean anterior left shank acceleration, maximum of medial-lateral first quartile of Fourier transform (FQFFT) for lower back, maximum of anterior-posterior FQFFT for lower back) achieved better classification results, with 77.3% accuracy, 66.1% sensitivity, 84.7% specificity, and 0.52 MCC score. All classification performance metrics improved when turn data was used for faller classification, compared to straight walking data. Combining turn and straight walking features decreased performance metrics compared to turn features for similar classifier model—feature selector combinations.

## 1. Introduction

Falls within elderly populations are a growing public health concern, with fatal and non-fatal fall injuries costing an estimated $23.3 billion in the United States, with a projected cost of $52 billion by 2020 [1,2]. Early fall risk detection and subsequent treatment are needed to mitigate fall incidence and improve quality of life for elderly individuals [3,4,5]. Wearable sensors that can be easily applied at the point-of-care [6] can facilitate quantitative assessments in clinical or older-adult care environments. Reviews of inertial-sensor applications for fall-risk classification in older-adults have recommended further research to determine if wearable sensors can be used to improve fall-risk prediction as a stand-alone assessment tool or supplement to clinical tests [7,8]. Combining appropriate wearable-sensor based features with machine learning techniques could advance fall-risk prediction tools and ultimately improve services for elderly people at risk of falling [6,9].

Fall risk prediction using clinical tests and wearable sensors has had variable success, with accuracy between 62 and 100%, specificity between 35 and 100%, and sensitivity between 55 and 99% [7]. While the top results are encouraging, the lower rates indicate a need for alternative methods to achieve consistently high outcomes. Fall risk assessment research has primarily focused on clinical tests, composed mainly of straight level walking, multiple tasks (e.g., sit-stand, walk-turn), and balance challenging tasks (e.g., stand on one leg, reach). Few quantitative studies involve more than straight walking, such as turns, to predict fall risk [10].

Turn-based features have been found to be important because of the decreased stability and increased energy expenditure when elderly individuals navigate turns [11,12,13,14]. Subtle fall-risk gait-based measures that may not be sensitive enough to reveal fall risk in straight walking may become highly effective fall-risk indicators when applied to walking turns. Elderly individuals at high risk of falling often perform a distinct turn (spin turn) compared to those at low risk of falling [15], suggesting a distinction between fallers and non-fallers for turn walking. A longer turn duration from the Timed Up and Go Test (TUG) [16] discriminated elderly fallers from non-fallers [17]. A study examining combinations of nine specific movements, turn time, and number of steps per turn discriminated between multiple fallers, non-multiple fallers, and able bodied individuals [18]. These clinical examinations of turns suggest that wearable-sensor based methods of assessing walking turns have good potential for classifying fallers.

Existing literature supports the hypothesis that turn data may contain information that can discriminate between fallers and non-fallers better than straight walking data. However, previous research using turns has focused on clinical assessment tools, temporal variables (completion time), or video analysis for fall risk prediction [18,19,20,21]. Faller classification research using wearable-sensor-based features from walking-turns is lacking. Furthermore, no comparison of straight and turn walking features for faller classification has been reported. Such a comparison would be useful for developing a better fall-risk assessment tool. Acceleration data acquired during the Six-Minute Walk Test (6MWT) [22] could provide both turn and straight walking information suitable for such an investigation. This paper presents a novel wearable-sensor based faller classification method, using walking-turn accelerometer-based features, and compares older-adult faller classification using straight and turn walking features. It is hypothesized that turn-walking based accelerometer features will provide better discriminating ability between prospective fallers and non-fallers, and thus provide better faller classification performance than corresponding straight-walking based features.

## 2. Materials and Methods

### 2.1. Participants

A convenience sample of 76 individuals, 65 years or older, (mean = 74.15 ± 7.0 years) were recruited from the community. Inclusion criteria were the ability to walk continuously and unaided for six minutes, no existing self-reported cognitive disorders, and not having experienced a fall during the six months prior to the study. Participants had a mean weight of 73.35 ± 13.4 kg and a mean height of 167.25 ± 10.0 cm. The study was approved by the University of Waterloo Research Ethics Committee on 1 August 2013, ORE #: 19106 and 30 June 2016, ORE #: 21599. All participants gave informed written consent.

### 2.2. Protocol

Participants reported their age and sex. Accelerometers (X16-1C, Gulf Coast Data Concepts, Waveland, MS, USA) were fitted to the posterior pelvis and left and right lateral shanks. Accelerometers were aligned with the vertical (upward: positive), medial-lateral (ML) (right: positive), and anterior-posterior (AP) axes (anterior: positive). Accelerometer data were collected with a nominal sampling rate of 50 Hz, (i.e., sampling rates varied slightly from 50 Hz for all accelerometers, as manufactured).

The six-minute walk test (6MWT) was conducted under standard conditions. Participants walked along a hallway, making consecutive left and right turns around two cones spaced 100 ft (30.34 m) apart [22]. Participants were instructed to alternate left and right turns around the cones until the end of the test and thus could not introduce bias into the turning direction.

A six-month follow-up fall-occurrence survey identified participants who fell at least once as prospective fallers (PF). All other participants were classified as non-fallers (NF). A fall was defined as an event that results in a person coming to rest unintentionally on the ground or other lower level, excluding falls from a stroke or overwhelming hazard [23].

Five participants were excluded because of accelerometer failure (two participants), unreliable data synchronization (one participant), incomplete prospective survey (one participant), and poor turn segmentation due to excessive noise between straight walking and turning sections (one participant). Therefore, 71 participants were included in the study, with 43 non-fallers and 28 prospective fallers.

### 2.3. Data Pre-Processing

Data for each accelerometer were imported into MATLAB 2014b (MathWorks, Natick, MA, USA) [24]. The sampling rates for each accelerometer differed slightly; therefore, all accelerometer signals were resampled to 50 Hz and then synchronized. This synchronization was performed using the first peak in vertical acceleration of each accelerometer.

**Figure 1:**
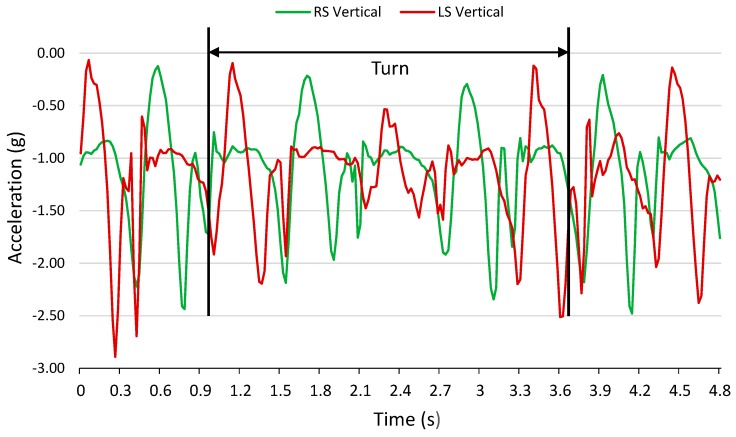
Vertical accelerometer signals from right shank (RS) and left shank (LS) with segmented turn.

**Figure 2:**
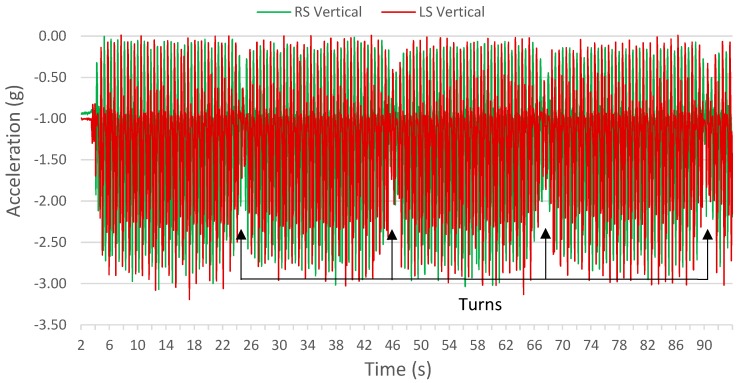
Section of left and right vertical accelerometer signals. Periodic drops in vertical acceleration magnitude locate turns in the 6MWT.

6MWT data were segmented into turn and straight sections. Turns were identified from a reduced magnitude in vertical accelerometer signal, defining the start of a turn (Figure 1). This periodic drop in vertical acceleration magnitude (Figure 2) was consistent with a turn occurring at the end of the 100 ft pathway that participants were instructed to walk on. The drop in vertical acceleration magnitude indicated a departure from the periodic straight section gait pattern; therefore, these sections were determined to be turns. In this paper, a turn was standardized as having five steps: a centre step and two adjacent steps on each side of the centre step. A 0.2 s buffer was added before and after the first and last steps. Multiple straight and turn sections were extracted from each 6MWT dataset. In all sections that follow, turn and straight walking data were treated independently, except during Test IV, described in Section 2.6.3.

### 2.4. Feature Extraction

A review of 40 inertial-sensor based fall risk studies found the dominant Fast Fourier Transform peak parameters (from lower-back accelerometers) and the ratio of even to odd harmonic (REOH) magnitudes (from head, upper back and lower back accelerometers) to both be recurring significant (*p* < 0.05) features when used to assess fall risk [7]. These features were carried forward in further research demonstrating their effectiveness for faller classification [25,26]. Temporal and acceleration descriptive statistics provided direct measures of body motion related to gait.

Accelerometer based features were calculated for each stride and then averaged across all strides, for each turn or straight section. Steps were identified by peak detection in the vertical acceleration signals. These peaks corresponded with foot strikes and were used in calculating the following accelerometer based features:

- Temporal: Cadence, stride time (time (s) from foot strike to the following foot strike of the same foot).
- Acceleration descriptive statistics: Acceleration maximum, mean, standard deviation for each direction for each of three axes (positive and negative of vertical, ML, and AP axes).
- Acceleration frequency: First quartile of Fourier transform (FQFFT) of each axis (vertical, medial-lateral, anterior-posterior). FQFFT is a percentage of acceleration frequencies within the first quartile (i.e., frequencies below 12.5 Hz) of an FFT frequency plot. A lower FQFFT value indicates the occurrence of more high frequency acceleration components during walking, which has been linked to instability [25,27,28].
- Ratio of even/odd harmonics (REOH): Ratio of acceleration signal in phase with stride frequency (inverse of stride time) [29,30,31]. Lower REOH values are associated with fall risk [29,30,32,33,34]. REOH was calculated for each axis (vertical, medial-lateral, anterior-posterior).

Twenty-four features were extracted for accelerometers: three descriptive statistics for each of three axes in both the positive and negative directions (3 × 3 × 2 = 18 features), FQFFT for three axes, and REOH for three axes. Cadence and stride time were calculated from acceleration measured by the lower-back accelerometer, for a total of 26 features for the lower back. Each straight and turn section had a total of 74 features (24 for left and right shanks, 26 for lower back: 24 + 24 + 26 = 74 features).

A single feature set was created for each participant using the maximum, minimum, standard deviation, and mean of the 74 features across all of a participant’s straight or turn sections. This produced a single feature set with 4 (maximum, minimum, mean, SD) × 74 (accelerometer derived features) = 296 features for each participant’s turn or straight data. Variation between steps and gait variability have been associated with fall risk [10,35]. Therefore, the standard deviation of repeated measurements of features across a test may be useful for faller classification. Extreme values of features (maxima or minima) have provided more useful information than mean values [36] and were therefore included with the mean and standard deviations.

### 2.5. Feature Selection

Classification difficulty may arise if many features are non-informative or redundant. These features can lead to poor model generalizability since the model may be modelling noise in the features, leading to poor classification results [37]. Feature selection was performed to eliminate redundant and non-informative features before classification [38]. Three feature selection methods (feature selectors) were used for each respective classifier to assess performance. The first feature selector, Select-k-Best, based on ANOVA F-statistics, selected features that accounted for the most variance between classes [39,40]. The variable k was set to 5 based on a heuristic search (select-5-best, S5B). The second feature selector (SEL) was based on Select False Positive Rate (SFPR) and Select False Discovery Rate (SFDR) methods, which chose features that minimized false positive and false discovery rates, respectively. The resulting list of SFPR and SFDR selected features were concatenated into a single non-redundant list. The number of features selected with SEL was not restricted. The third feature selector, recursive feature elimination (RFE), performed multiple data classifications using a random forest classifier, kept features that provided better classification results, and eliminated features with poorer results [41,42]. This process was repeated until the five best features were selected. Feature selection was performed only on training data for the classifier models. The selected features were then applied to the testing data for classification. Division and use of training and testing datasets are described in Section 2.6.2.

### 2.6. Classification

#### 2.6.1. Machine Learning Models

Six classifier models were trained to classify participants as faller or non-faller: two *k*-nearest neighbor (*k*NN) classifiers with *k* = 3 (3NN) and *k* = 5 (5NN); three support vector machines (SVM) with linear, third, and fifth order polynomial kernels; and one random forest (RF) model. RF and *k*NN are non-parametric models that allow irregular class boundaries. All SVMs used a method where overlapping classes may become separable by using the “kernel trick” by projecting the data into higher dimensions [43,44]. RF is an ensemble method that creates a strong classifier based on many decision trees, thereby accommodating individual tree weaknesses. One hundred decision trees were trained for each RF classifier. Models were generated with the Scikit-Learn library [41].

#### 2.6.2. Cross Validation

A subset of the full dataset was used for model training, and the remaining data subset (testing dataset) was used to evaluate model performance for all faller-classification tests. Two cross validation (CV) methods were used (the sequence of tests is described in Section 2.6.3): five-fold cross validation (5FCV) and 2500-iteration random-shuffle-split cross validation (2500-RSS). Both methods used stratified data splits, which ensured that the ratio of fallers to non-fallers from the whole dataset was preserved in both the training and testing data.

5FCV divided the data into five stratified subsets (20% data in each subset), with one subset chosen for model testing and the remaining four subsets combined for model training. The three feature selectors (Select-*k*-Best, SEL and RFE) were applied to the training subset, thereby providing three best feature sets for classification. Classifier training (on four subsets combined) and testing (on the fifth subset) were then performed five times such that every subset was used as the testing set. The five sets of results were averaged to obtain final results for each classification model—feature-selector combination. With six classifier methods and three feature selection methods, a total of 18 classification-model—feature-selector (CM-FS) combinations were generated from 5FCV. The best CM-FS combinations were used in the 2500-RSS for both straight and turn-based data.

For 2500-RSS, a single stratified-random-shuffle split was configured to select a stratified random subset of 80% of the data for training the model with the remaining 20% of the data as a stratified random subset for model testing. This process was repeated for 2500 iterations. For each iteration, feature selection was performed on the training data and a new classification model was trained and tested. Feature selection was based solely on cross validation iteration training data. Mean, standard deviation, and confidence interval were calculated based on results from the 2500 iterations. Unlike 5FCV, this method does not guarantee that all testing subsets will be disjoint. However, because of the large number of iterations, many unique data splits will determine if the models generalize well. The chosen number of iterations was based on convergence of the classifier mean accuracy.

Within each cross-validation described above, normalization of features was performed before feature selection and classifier training. Normalization of features allows faster model training [38,45]. Each feature value in a participant’s feature set was normalized to the range [0, 1] as follows:

(1) $$y_{normalized}=\frac{y-y_{min}}{y_{max}-y_{min}},$$

where *y* is a feature value from one participant, and *y_min_* and *y_max_* are the minimum and maximum values of that feature, respectively, across all participants within a training set for each cross-validation fold. These normalization parameters, *y_min_* and *y_max_* from the training set, were used to normalize the testing data features. This normalization prevents testing data from biasing classifier training.

#### 2.6.3. Performance Evaluation

Performance for each CM-FS combination was evaluated using accuracy (ACC), specificity (SPEC), sensitivity (SENS), negative predictive value (NPV), positive predictive value (PPV), F1 score, and Matthews Correlation Coefficient (MCC) [46,47]. For 5FCV, means for these metrics were calculated over the five cross-validation folds. For 2500-RSS, mean, standard deviation and confidence interval of these metrics were calculated over the 2500 iterations.

To determine the best performing CM-FS combination, classifier performance metrics were sorted in descending order with the largest result (best) given a value of 1, the second a 2, etc. Ties were given the same rank, with the next non-tied classifier being ranked by their position after accounting for the tied classifiers (e.g., a three-way tie at position three results in: 1, 2, 3, 3, 3, 6, 7, …) [48]. Rankings were summed across performance measures, with the lowest sum indicating the best classifier. This generated one score for each CM-FS combination.

**Figure 3:**
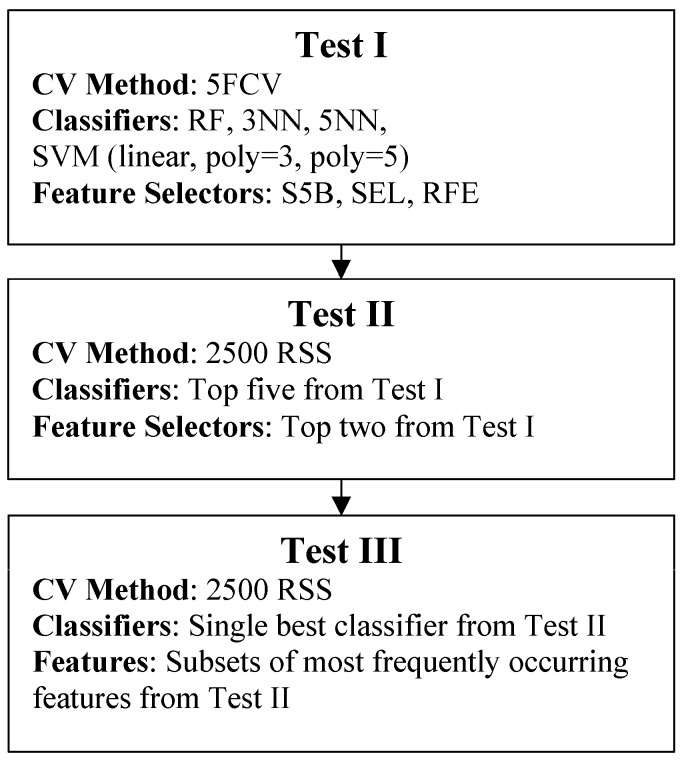
Model performance evaluations.

**Figure 4:**
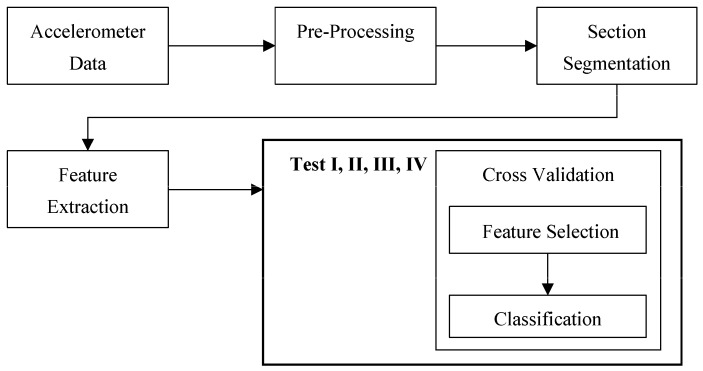
Overview of data processing and classification process.

Three tests were performed in sequence for both straight and turn data separately (Figure 3), Test I performed first, then Test II, followed by Test III. A final test, Test IV, was performed using all turn and straight features together to determine if including all features would further improve or worsen performance. An overview of the flow of data and classification methods is shown in Figure 4. Test I used 5FCV for all 18 CM-FS combinations (six classifiers, three feature selectors). The top-nine combinations were evaluated and one classifier and one feature selector that appeared the least were discarded, for both straight and turn results, which expedited training. The 10 remaining CM-FS combinations were then used in Test II. Test II used 2500 RSS cross validation to evaluate performance of the remaining five classifiers and two feature selectors combinations (10 CM-FS combinations). Welch’s *t*-tests compared straight and turn performance metrics for the best straight and turn based CM-FS combinations from Test II.

**Figure 5:**
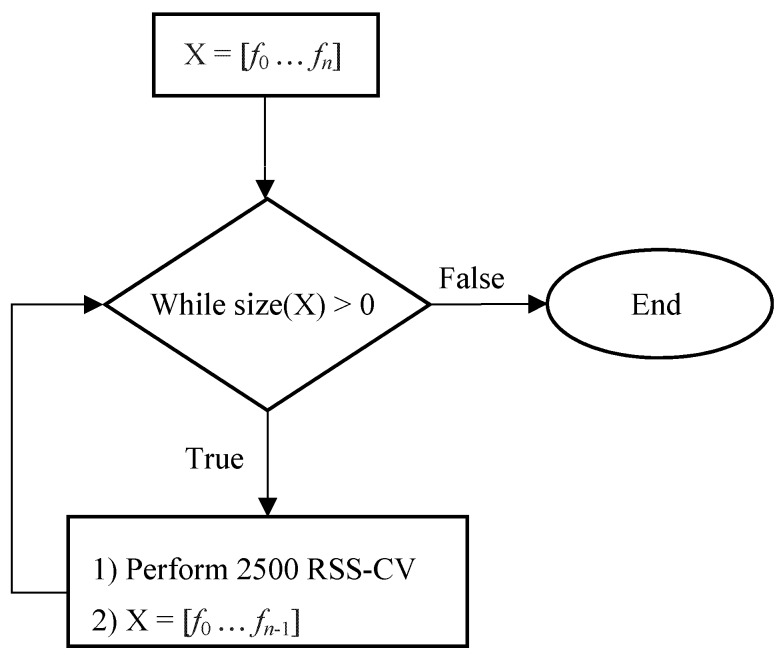
Test III procedure for testing most frequently occurring feature subsets.

For Test III, the most frequently occurring (MFO) features from the feature selections of Test II, selected for 250 or more iterations (selected for 10% of the iterations from 2500-RSS cross validation), were combined into multiple sets. The entire set of most frequent features was ordered from most frequent (*f*_0_) to least frequent (*f_n_*), X_0_ = [*f*_0_ … *f_n_*]. The first set was composed of all of the most frequent features, X_0_ = *f*_0_ … *f_n_*], the second set was composed of the *n* − 1 most frequent features, X_1_ = [*f*_0_ … *f_n_*_− 1_], the third set was composed of the *n* − 2 most frequent features, X_2_ = [*f*_0_ *… f_n_*_− 2_], and so on until the final subset had only the most frequent feature X*_n_* = [*f*_0_]. Starting with a set of all the most frequent features to a final set having one feature, 2500-RSS cross validation was performed for each new generated feature set X*_i_*, *i* = [0, *n*] (Figure 5), using the best classifier model from Test II. This analysis was performed for straight and turn data. Test III determined the best subsets of features for faller classification.

For Test IV, a combined feature set, composed of all straight and turn based features, was used with the top four best performing classifier-models from Test II and the best two feature selectors from Test I, to provide a set of CM-FS combinations. Before feature selection, the feature set of each participant had a concatenation of all straight and turn features, a total of 592 features (2 × 296). A 2500 RSS cross validation was performed to evaluate the classification performance of the combined straight and turn feature set with the selected CM-FS combinations.

To promote classification generalizability and reliability, and to avoid methodological problems associated with validation and training-testing protocols seen in the fall-risk assessment literature [49], two stratified cross-validation methods were used. The top classifiers and feature selectors were chosen in Test I using 5FCV and then used for Test II, which used 2500-RSS cross validation.

## 3. Results

### 3.1. Test I

Test I results for straight-walking using 5FCV are presented in Table 1. The RF and S5B combination was the best with 62.0% accuracy, 46.4% sensitivity, 72.1% specificity and 0.19 MCC. The second-best model also used S5B feature selection, and had greater sensitivity (78.6%) but lower specificity and accuracy.

Compared to straight walking, turn data had better faller classification (Table 2). The best turn-based combination was RF S5B, with 77.5% accuracy, 67.9% sensitivity, 83.7% specificity, and 0.52 MCC score. The second best results, obtained using RF SEL, were similar to RF S5B.

RF, 3NN, and 5NN, and linear and third order polynomial SVM classifiers performed best in Test I. The worst performing classifier was the fifth degree polynomial SVM, which appeared only once in the top-nine combinations for the straight data and not at all for the turn data. S5B and SEL feature selectors performed better than RFE using the same classifier models. The worst feature selector was the RFE, which appeared four times, compared to seven times for S5B and SEL methods. Based on these results, the fifth order polynomial SVM classifier and RFE selector were eliminated from further tests. Therefore, RF, 3NN, 5NN, and linear and third order polynomial SVM classifiers, and S5B and SEL feature selectors were used for Test II, for both turn and straight datasets.

### 3.2. Test II

#### 3.2.1. Classification

Faller classification results for Test II (Table 3 and Table 4) were similar to Test I. Faller classification with turn data (Table 4) outperformed straight walking data (Table 3). The best turn-based combination (RF S5B) had 73.4% accuracy, 60.5% sensitivity, 82.0% specificity, and 0.44 MCC score. The best straight-walking-based combination (3NN S5B) had 55.5% accuracy, 46.1% sensitivity, 61.8% specificity and 0.08 MCC score. All performance metrics (accuracy, sensitivity, specificity, PPV, NPV, F1-score, and MCC) of the best turn-feature based CM-FS combination (RF S5B) were significantly greater (*p* < 0.001) than the corresponding metrics of the best straight-feature based CM-FS combination (3NN S5B).

#### 3.2.2. Selected Features

As described in Section 2.4, a single feature set was created for each participant using the maximum, minimum, standard deviation (SD), and mean of the 74 features across all of a participant’s straight sections, and similarly a single feature set was created based on all turn sections.

**Figure 6:**
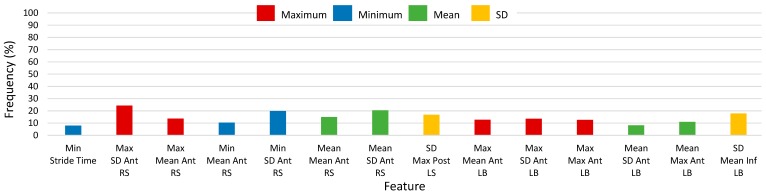
Histogram of selected straight-walking feature frequency above 8% (200) of 2500 total selections using the combination of Select False Positive Rate and Select False Discovery Rate methods (SEL) for 2500 random-shuffle-split iterations.

**Figure 7:**
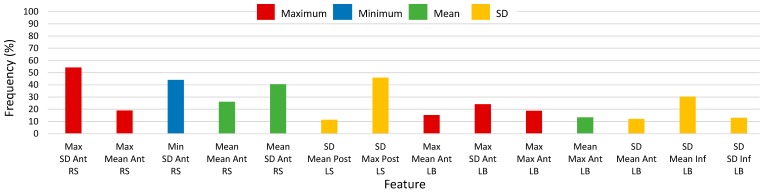
Histogram of selected straight-walking feature frequency above 8% (200) of 2500 total selections using the select-5-best (S5B) method for 2500 random-shuffle-split iterations.

Histograms of 2500-RSS selected features with selection frequencies above 8% (200 out of 2500 iterations) for straight walking, using SEL and S5B, are shown in Figure 6 and Figure 7, respectively (note that “MFO features” include only features with selection frequency above 250). The most frequently occurring S5B features, in descending order of frequency, were: maximum of SD of anterior RS acceleration, SD of maximum posterior LS acceleration, minimum of SD of anterior RS acceleration, mean of SD anterior RS acceleration, SD of mean inferior LB acceleration, mean of mean anterior RS acceleration, maximum of SD anterior LB acceleration, maximum of mean anterior RS acceleration, maximum of maximum anterior LB acceleration, maximum of mean anterior LB acceleration, mean of maximum anterior LB acceleration, SD of SD inferior LB acceleration, SD of mean anterior LB acceleration, SD of mean posterior LS acceleration. For the SEL method, the top features were similar; however, SEL frequencies were lower overall and frequency ordering was not the same.

**Figure 8:**
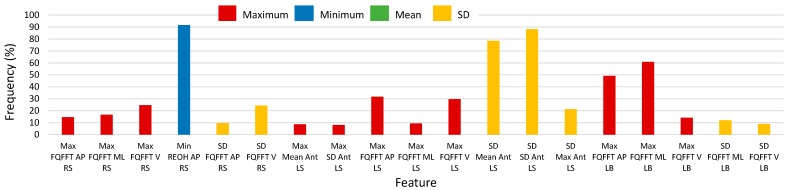
Histogram of selected turn-based feature frequency above 8% (200) of 2500 total selections using the combination of Select False Positive Rate and Select False Discovery Rate methods (SEL) for 2500 random-shuffle-split iterations.

**Figure 9:**
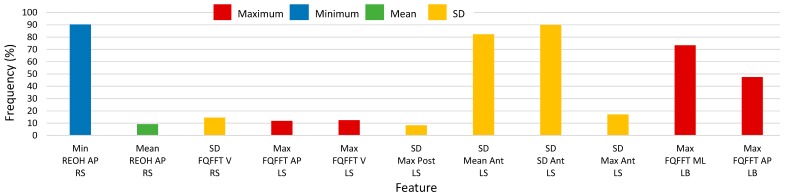
Histogram of selected turn-based feature frequency above 8% (200) of 2500 total selections using the select-5-best (S5B) method for 2500 random-shuffle-split iterations.

Histograms of 2500-RSS selected features with selection frequencies above 8% (200 out of 2500 iterations) for turns, using SEL and S5B, are shown in Figure 8 and Figure 9, respectively (note that “MFO features” include only features with selection frequency above 250). The most frequently occurring turn based features for the S5B method, in descending order of frequency, were: minimum of anterior-posterior REOH for RS, SD of SD anterior LS acceleration, SD of mean anterior LS acceleration, maximum of medial-lateral FQFFT for LB, maximum of anterior-posterior FQFFT for LB, SD of maximum anterior LS acceleration, SD of vertical FQFFT for RS, maximum of vertical FQFFT for LS, and maximum of anterior-posterior FQFFT for LS. For the SEL method, the top features were similar; however, frequency ordering was slightly different.

### 3.3. Test III

The best results for straight walking (Table 5) were for the 5 MFO feature subset (maximum of SD of anterior RS acceleration, SD of maximum posterior LS acceleration, minimum of SD of anterior RS acceleration, mean of SD anterior RS acceleration, SD of mean inferior LB acceleration), with 64.1% accuracy, 59.9% sensitivity, 66.9% specificity, and 0.26 MCC score. For turn walking (Table 6), the best results were for the 5 MFO feature subset (minimum of anterior-posterior REOH for RS, SD of SD anterior LS acceleration, SD of mean anterior LS acceleration, maximum of medial-lateral FQFFT for LB, maximum of anterior-posterior FQFFT for LB), with 77.3% accuracy, 66.1% sensitivity, 84.7% specificity, and 0.52 MCC score. The Test III results were generally superior to those of Test II, where all accuracies of Test III were greater than those for Test II.

### 3.4 Test IV

#### 3.4.1. Classification

The best classification results for the combined set of straight and turn based features (Table 7) were attained using a RF S5B combination, with 71.6% accuracy, 57.5% sensitivity, 81.1% specificity and 0.4 MCC score. The best three CM-FS combinations for Test IV were the same as the best turn-based feature CM-FS combinations in Test II. The CM-FS combinations from Test IV (combined straight and turn based feature sets) provided better performance metrics than the corresponding straight-based feature CM-FS combinations in Test II, and were similar or slightly worse performance metrics than for the corresponding turn-based feature CM-FS combinations in Test II.

#### 3.4.2. Selected Features from Combined Straight and Turn Feature Set

The S5B and SEL methods selected similar MFO features. For both methods, nine of the ten MFO features selected from the combined straight and turn feature set were turn-based features. These nine features were the same as the turn-based MFO features from Test II. These included: the minimum of anterior-posterior REOH for RS, SD of SD anterior LS acceleration, SD of mean anterior LS acceleration, maximum of medial-lateral FQFFT for LB, maximum of anterior-posterior FQFFT for LB, SD of maximum anterior LS acceleration, SD of vertical FQFFT for RS, maximum of vertical FQFFT for LS, and maximum of anterior-posterior FQFFT for LS. The only straight walking feature among the 10 MFO features was the SD of maximum posterior LS acceleration using the S5B algorithm, and the maximum of SD of anterior RS acceleration for the SEL algorithm.

## 4. Discussion

A new method for faller classification in older adults was developed using walking-turn accelerometer-based features extracted from wearable sensor data. This research confirmed that turn features performed better than straight walking features for prospective faller classification, and the best overall classification method used a random forest classifier and five turn-based features, obtained from the S5B feature selection process.

Test I determined that turn features performed better than straight walking features for prospective faller classification since turn-based models had greater accuracy, sensitivity, specificity, F1-score, and MCC than straight-walking models. Test II reinforced the conclusions from Test I, since turn features also outperformed straight walking features for faller classification. The best turn-based classifier-feature selector combination (RF-S5B) had results that were at least 24% greater than corresponding best straight-walking results, with the worst turn-based classifier outperforming the best straight-walking-based classifier. All performance metrics of the best turn-feature based CM-FS combination were significantly greater than the corresponding metrics of the best straight-feature based CM-FS combination. The narrow confidence intervals, which were less than ±1% for turn classification performance metrics and ±1.32% for straight walking, support the generalizability of these results for population-based applications. Based on the law of large numbers [50] and narrow 95% confidence intervals, the 2500-RSS, used for Tests II and III, generated viable mean results, indicating that the mean values were likely similar to population values.

Test III, using 2500-RSS cross validation, again confirmed the findings that turn features produced a better performing classifier than straight-walking based features. Test III also determined that, for turns, the best feature subset included minimum of anterior-posterior REOH for right shank, SD of SD anterior left shank acceleration, SD of mean anterior left shank acceleration, maximum of medial-lateral FQFFT for lower back, and maximum of anterior-posterior FQFFT for lower back. Feature maxima, minima, and SD appeared more often in the best feature subset than mean-based features. This suggested that extreme values (maximum and minimum) and variability (SD) provide better discriminative information for turns, as found in previous research [36].

Test IV was performed to determine if all available features from both straight and turn sections would further improve performance over turn-only-based features. Poorer performance was observed for the CM-FS combinations of Test IV (straight and turn features) compared to their corresponding turn-only-based feature CM-FS combinations from Test II. This suggests that adding straight-walking-based features does not aid in faller classification when turn-based features are used. Furthermore, it was found that during the 2500 iterations of feature selection for both S5B and SEL methods, nine of the ten MFO features were turn-based features, showing that the information in the turn-based features was more useful for classification.

The most frequently occurring turn feature in the feature selection process (Test II and Test IV) was minimum anterior-posterior REOH for the right shank, which composed the 1 MFO feature subset. Interestingly, only modest differences occurred between the 1 MFO feature subset and the best feature subset (5 MFO Feature). The strong performance using only the minimum-AP-REOH-right-shank feature indicates the importance of this feature for faller classification. This result is supported by [25,29,30,32,33,34,51], where a small REOH indicated step-to-step asymmetry within strides and possibly gait instability. Two features in the 5 MFO feature subset involved the lower back sensor maximum FQFFT, across all turn sections for the anterior-posterior and medial-lateral axes. A low FQFFT value indicates more high frequency than low frequency components. Walking can be associated with activities linked to decreased stability [52] and higher frequency components indicate less steady movements [27,28] and possibly sudden movements to recover balance; therefore, frequency components at the lower back may be useful for faller classification. The remaining two features of the 5 MFO feature subset were the SD of the mean anterior left shank acceleration and SD of anterior left shank acceleration SD. These features were related to variation across different sections, suggesting that acceleration variation over time can be a good indicator for faller classification. More gait variability has been linked to fall risk [10,35]. To enable further interpretation of the discriminative ability of the 5 MFO feature subset, a statistical comparison of faller and non-faller group feature values should be undertaken in a future study.

Previous approaches that used turn-walking to discriminate fallers and non-fallers mainly used the TUG test [18,19,20,21,27]. However, a meta-analysis of 53 studies suggested that TUG was ineffective for determining fall risk for healthy older individuals [53]. This was primarily due to variations in the thresholds across studies used to classify fallers and non-fallers. Since this study included multiple turn sections and found that classification using turn-based features performed better than using straight-walking features, the methods of this study may be a more suitable alternative than the TUG for prospectively classifying fallers.

Other faller classification studies have found better and worse classification results compared to this paper, with accuracies between 62 and 100%, specificities between 35 and 100%, and sensitivities between 55 and 99% [7] based on straight walking data. The types of populations (retrospective or prospective fallers; single-fall or multiple-fall fallers) and methodologies vary, and differ from the current paper. The prospective fall prediction study in [26] permits comparison based on the identical older-adult population. The turn-feature based classification results in this paper (73.4% accuracy, 60.5% sensitivity, 82.0% specificity, and 0.44 MCC score) were better than the best straight-walking classification results in [26] (56.5% accuracy, 42.5% sensitivity, 65.4% specificity and 0.083 MCC score), based on similar accelerometer derived features for 25 ft walk single-task and dual-task tests, and similar cross validation with 10,000 random stratified splits. Those results for straight walking were similar to the straight-walking-based classification results in Test II of this paper (55.5% accuracy, 46.1% sensitivity, 61.8% specificity and 0.08 MCC). Since the straight-walking classifier performances for [26] and this paper were similar, it is likely that the use of turn-based features was the main contributing factor to improved classification results (turn compared to straight), rather than the inclusion of more walking sections (6MWT in this paper compared to 25 ft walk test in [26]). This strongly suggests that turn-based features provide better information for prospective faller classification and thus faller prediction.

In this study, a turn was defined using a fixed number of steps. While this standardized the analysis, this method may have led to one or two extra or missed steps for a participant’s turn. The effect on the REOH feature from fixing the number of steps in a turn to five is unknown and would be of interest for further study. Turn segmentation could be improved using gyroscope data or video capture of the walking trial.

Existing elderly fall screening assessments could benefit by better prospective faller classification. The results of this research suggest that integrating wearable-sensor turn-based features and machine learning in elderly screening assessments may improve faller identification. Since a shorter test might be easier to administer in a clinical setting, future research could study whether a shorter distance with fewer turns could also be effective. This study has demonstrated that turn-based features permit better prospective faller classification than using straight-walking features. Future studies employing improved turn segmentation or turning tasks (e.g., figure-eight patterns) without need for segmentation, and additional features (e.g., entropy [54], frequency based) could lead to more reliable classification suitable for clinical implementation.

## 5. Conclusions

A novel wearable-sensor based faller classification method using walking-turns was developed. This work is the first to directly compare prospective classification results using straight and turn walking data, based on wearable-accelerometer measures. A marked improvement in all classification performance metrics occurred when turn data was used for faller classification, compared to straight walking data. Turn data acquired from accelerometers contains useful biomechanical information that can improve prospective fall risk classification for healthy older adults. A random forest classifier paired with a select-5-best (S5B) feature selector provided the best classification results for both turn and straight walking data. The most frequently occurring turn feature in the feature selection process was the minimum anterior-posterior REOH for the right shank, which formed the 1 MFO feature subset and produced comparable results to the 5 MFO feature subset, indicating the importance of this feature for faller classification. Future work could examine the effectiveness of the most frequently selected, best performing turn features on faller classification in other populations. Combining straight and turn-based features for prospective faller classification did not improve classification models that used only turn-based features.

## Figures and Tables

**Table 1:** Straight-walking section five-fold cross validation (5FCV) results. PPV: positive predictive value, NPV: negative predictive value, MCC: Matthews correlation coefficient, S5B: select-5-best, SEL: false positive and discovery rate method, RFE: recursive feature eliminator, RF: random forest, *k*NN: *k*-nearest neighbour, SVM: support vector machine, linear: linear kernel, poly: polynomial kernel.

Classifier, Feature Selector	Accuracy (%)	Sensitivity (%)	Specificity (%)	PPV (%)	NPV (%)	F1 Score	MCC	Rank Sum
RF S5B	62.0	46.4	72.1	52.0	67.4	0.49	0.19	24
SVM (poly = 3) S5B	56.3	78.6	41.9	46.8	75.0	0.59	0.21	26
RF SEL	57.7	46.4	65.1	46.4	65.1	0.46	0.12	36
RF RFE	62.0	32.1	81.4	52.9	64.8	0.40	0.16	44
SVM (poly = 5) SEL	54.9	57.1	53.5	44.4	65.7	0.50	0.10	46
SVM (poly = 3) RFE	52.1	71.4	39.5	43.5	68.0	0.54	0.11	47
SVM (poly = 3) SEL	52.1	71.4	39.5	43.5	68.0	0.54	0.11	47
*k*NN (*k* = 5) SEL	56.3	42.9	65.1	44.4	63.6	0.44	0.08	51
*k*NN (*k* = 3) S5B	54.9	50.0	58.1	43.8	64.1	0.47	0.08	51
*k*NN (*k* = 3) SEL	56.3	39.3	67.4	44.0	63.0	0.42	0.07	60
SVM (linear) S5B	53.5	50.0	55.8	42.4	63.2	0.46	0.06	63
SVM (linear) SEL	53.5	50.0	55.8	42.4	63.2	0.46	0.06	64
SVM (poly = 5) RFE	52.1	46.4	55.8	40.6	61.5	0.43	0.02	78
SVM (linear) RFE	52.1	39.3	60.5	39.3	60.5	0.39	0.00	85
*k*NN (*k* = 3) RFE	50.7	35.7	60.5	37.0	59.1	0.36	−0.04	97
SVM (poly = 5) S5B	49.3	39.3	55.8	36.7	58.5	0.38	−0.05	100
*k*NN (*k* = 5) S5B	47.9	35.7	55.8	34.5	57.1	0.35	−0.08	109
*k*NN (*k* = 5) RFE	46.5	35.7	53.5	33.3	56.1	0.34	−0.11	119

**Table 2:** Turn section five-fold cross validation (5FCV) results. PPV: positive predictive value, NPV: negative predictive value, MCC: Matthews correlation coefficient, S5B: select-5-best method, SEL: false positive and discovery rate method, RFE: recursive feature eliminator, RF: random forest, *k*NN: *k*-nearest neighbour, SVM: support vector machine, linear: linear kernel, poly: polynomial kernel.

Classifier, Feature Selector	Accuracy (%)	Sensitivity (%)	Specificity (%)	PPV (%)	NPV (%)	F1 Score	MCC	Rank Sum
RF S5B	77.5	67.9	83.7	73.1	80.0	0.70	0.52	12
RF SEL	77.5	64.3	86.0	75.0	78.7	0.69	0.52	14
RF RFE	69.0	53.6	79.1	62.5	72.3	0.58	0.34	38
*k*NN (*k* = 5) SEL	69.0	50.0	81.4	63.6	71.4	0.56	0.33	45
*k*NN (*k* = 5) S5B	71.8	42.9	90.7	75.0	70.9	0.55	0.39	46
SVM (linear) S5B	67.6	53.6	76.7	60.0	71.7	0.57	0.31	50
SVM (linear) SEL	66.2	57.1	72.1	57.1	72.1	0.57	0.29	55
*k*NN (*k* = 3) S5B	67.6	50.0	79.1	60.9	70.8	0.55	0.30	57
SVM (poly = 3) RFE	62.0	67.9	58.1	51.4	73.5	0.58	0.25	58
*k*NN (*k* = 3) SEL	66.2	50.0	76.7	58.3	70.2	0.54	0.28	70
SVM (poly = 3) SEL	60.6	64.3	58.1	50.0	71.4	0.56	0.22	73
SVM (poly = 5) SEL	54.9	78.6	39.5	45.8	73.9	0.58	0.19	76
SVM (poly = 5) S5B	60.6	60.7	60.5	50.0	70.3	0.55	0.21	81
*k*NN (*k* = 5) RFE	63.4	35.7	81.4	55.6	66.0	0.43	0.19	89
SVM (linear) RFE	60.6	50.0	67.4	50.0	67.4	0.50	0.17	94
SVM (poly = 3) S5B	59.2	57.1	60.5	48.5	68.4	0.52	0.17	97
*k*NN (*k* = 3) RFE	62.0	39.3	76.7	52.4	66.0	0.45	0.17	98
SVM (poly = 5) RFE	57.7	46.4	65.1	46.4	65.1	0.46	0.12	114

**Table 3:** Straight-walking section results for 2500-iteration random-shuffle-split cross validation (2500-RSS), ordered by ranked performance. PPV: positive predictive value, NPV: negative predictive value, MCC: Matthews correlation coefficient, S5B: select-5-best method, SEL: false positive and discovery rate method, RFE: recursive feature eliminator, RF: random forest, *k*NN: *k*-nearest neighbour, SVM: support vector machine, linear: linear kernel, poly: polynomial kernel, $\bar{x}$: mean, SD: standard deviation, CI: 95% confidence interval.

Classifier, Feature Selection	Accuracy (%)			Sensitivity (%)			Specificity (%)			PPV (%)			NPV (%)			F1			MCC		
	$\bar{x}$	SD	CI	$\bar{x}$	SD	CI	$\bar{x}$	SD	CI	$\bar{x}$	SD	CI	$\bar{x}$	SD	CI	$\bar{x}$	SD	CI	$\bar{x}$	SD	CI
*k*NN (*k* = 3) S5B	55.5	12.0	0.47	46.1	21.2	0.83	61.8	16.2	0.64	44.6	16.9	0.66	63.2	11.5	0.45	0.45	0.17	0.007	0.08	0.26	0.010
RF S5B	56.2	11.4	0.45	39.8	20.3	0.80	67.2	15.9	0.62	44.7	19.6	0.77	62.6	9.9	0.39	0.42	0.18	0.007	0.07	0.26	0.010
RF SEL	56.9	11.2	0.44	34.5	20.3	0.79	71.9	18.3	0.72	45.0	25.2	0.99	62.2	8.7	0.34	0.39	0.18	0.007	0.07	0.30	0.012
SVM (poly = 3) SEL	51.7	11.1	0.43	59.7	33.6	1.32	46.4	30.3	1.19	42.6	18.8	0.74	63.3	25.6	1.00	0.50	0.20	0.008	0.06	0.39	0.015
*k*NN (*k* = 5) S5B	55.0	11.8	0.46	43.6	21.8	0.85	62.7	17.0	0.67	43.8	18.3	0.72	62.5	11.2	0.44	0.44	0.18	0.007	0.06	0.26	0.010
SVM (linear) SEL	53.4	12.1	0.48	50.3	23.7	0.93	55.5	23.7	0.93	43.0	17.3	0.68	62.6	15.9	0.62	0.46	0.16	0.006	0.06	0.30	0.012
SVM (linear) S5B	50.9	11.9	0.47	53.6	25.4	0.99	49.1	19.3	0.76	41.3	15.0	0.59	61.4	16.4	0.64	0.47	0.17	0.007	0.03	0.27	0.011
*k*NN (*k* = 3) SEL	54.0	11.4	0.45	37.5	19.9	0.78	65.1	17.3	0.68	41.7	19.6	0.77	61.0	9.7	0.38	0.39	0.17	0.007	0.03	0.25	0.010
SVM (poly = 3) S5B	48.7	10.4	0.41	61.6	33.6	1.32	40.1	26.2	1.03	40.7	16.1	0.63	61.0	26.4	1.03	0.49	0.20	0.008	0.02	0.35	0.014
*k*NN (*k* = 5) SEL	53.8	10.8	0.42	34.6	19.9	0.78	66.6	17.7	0.69	40.8	20.5	0.80	60.4	9.2	0.36	0.37	0.17	0.007	0.01	0.26	0.010

**Table 4:** Turn section results for 2500-iteration random-shuffle-split cross validation (2500-RSS), ordered by ranked performance. PPV: positive predictive value, NPV: negative predictive value, MCC: Matthews correlation coefficient, S5B: select-5-best method, SEL: false positive and discovery rate method, RFE: recursive feature eliminator, RF: random forest, *k*NN: *k*-nearest neighbour, SVM: support vector machine, linear: linear kernel, poly: polynomial kernel, $\bar{x}$: mean, SD: standard deviation, CI: 95% confidence interval.

Classifier, Feature Selector	Accuracy (%)			Sensitivity (%)			Specificity (%)			PPV (%)			NPV (%)			F1			MCC		
	$\bar{x}$	SD	CI	$\bar{x}$	SD	CI	$\bar{x}$	SD	CI	$\bar{x}$	SD	CI	$\bar{x}$	SD	CI	$\bar{x}$	SD	CI	$\bar{x}$	SD	CI
RF S5B	73.4	10.6	0.42	60.5	20.5	0.81	82.0	12.8	0.50	69.1	18.2	0.71	75.7	10.2	0.40	0.65	0.17	0.007	0.44	0.24	0.009
RF SEL	71.6	10.9	0.43	58.3	20.7	0.81	80.4	13.3	0.52	66.5	18.5	0.72	74.3	10.3	0.41	0.62	0.17	0.007	0.40	0.24	0.010
*k*NN (*k* = 5) S5B	69.2	11.2	0.44	49.0	21.4	0.84	82.7	13.3	0.52	65.3	22.5	0.88	70.8	9.7	0.38	0.56	0.19	0.008	0.34	0.27	0.011
*k*NN (*k* = 3) S5B	68.0	11.2	0.44	50.8	20.7	0.81	79.6	13.9	0.55	62.4	20.9	0.82	70.8	9.8	0.39	0.56	0.18	0.007	0.32	0.26	0.010
SVM (linear) S5B	66.7	11.7	0.46	57.6	20.8	0.82	72.8	16.0	0.63	58.5	17.7	0.69	72.0	11.7	0.46	0.58	0.16	0.006	0.30	0.25	0.010
SVM (linear) SEL	64.7	13.0	0.51	57.6	24.2	0.95	69.5	17.9	0.70	55.7	19.3	0.76	71.1	14.4	0.56	0.57	0.19	0.007	0.27	0.31	0.012
*k*NN (*k* = 5) SEL	67.2	12.5	0.49	48.7	21.6	0.85	79.5	14.9	0.58	61.3	23.2	0.91	69.9	10.5	0.41	0.54	0.20	0.008	0.30	0.29	0.012
*k*NN (*k* = 3) SEL	66.8	12.7	0.50	50.0	21.3	0.83	78.0	15.2	0.60	60.3	22.2	0.87	70.1	10.8	0.42	0.55	0.19	0.008	0.29	0.29	0.011
SVM (poly = 3) SEL	61.8	13.1	0.51	50.7	25.3	0.99	69.2	24.8	0.97	52.3	25.1	0.98	67.8	15.4	0.61	0.51	0.18	0.007	0.20	0.33	0.013
SVM (poly = 3) S5B	60.7	13.8	0.54	55.7	23.6	0.93	64.1	22.3	0.87	50.8	20.0	0.78	68.4	15.8	0.62	0.53	0.17	0.007	0.20	0.30	0.012

**Table 5:** Most frequently occurring (MFO) feature subsets for straight-walking section results and 3NN classifier using 2500-iteration random-shuffle-split cross validation (2500-RSS), ordered by ranked performance. PPV: positive predictive value, NPV: negative predictive value, MCC: Matthews correlation coefficient, $\bar{x}$: mean, SD: standard deviation, CI: 95% confidence interval.

# Features	Accuracy (%)			Sensitivity (%)			Specificity (%)			PPV (%)			NPV (%)			F1			MCC		
	$\bar{x}$	SD	CI	$\bar{x}$	SD	CI	$\bar{x}$	SD	CI	$\bar{x}$	SD	CI	$\bar{x}$	SD	CI	$\bar{x}$	SD	CI	$\bar{x}$	SD	CI
5	64.1	10.8	0.42	59.9	19.2	0.75	66.9	14.5	0.57	54.7	14.3	0.56	71.4	11.0	0.43	0.57	0.14	0.006	0.26	0.23	0.009
3	63.1	11.3	0.44	61.2	20.0	0.78	64.4	14.9	0.59	53.4	14.0	0.55	71.3	11.9	0.47	0.57	0.15	0.006	0.25	0.24	0.009
4	62.2	10.8	0.42	57.7	18.9	0.74	65.2	15.0	0.59	52.5	14.6	0.57	69.8	10.7	0.42	0.55	0.14	0.006	0.23	0.23	0.009
9	61.5	10.4	0.41	42.1	18.8	0.74	74.5	14.0	0.55	52.4	20.0	0.79	65.9	8.5	0.33	0.47	0.17	0.007	0.17	0.24	0.009
10	60.7	11.1	0.43	44.7	19.9	0.78	71.4	14.6	0.57	51.1	19.1	0.75	66.0	9.5	0.37	0.48	0.17	0.007	0.17	0.25	0.010
6	60.6	12.3	0.48	56.1	20.1	0.79	63.6	16.7	0.66	50.7	16.0	0.63	68.5	12.2	0.48	0.53	0.16	0.006	0.20	0.26	0.010
2	60.0	11.5	0.45	57.2	19.3	0.76	61.8	15.9	0.62	50.0	14.5	0.57	68.4	11.7	0.46	0.53	0.15	0.006	0.19	0.24	0.009
8	60.6	10.3	0.40	38.5	18.7	0.73	75.4	13.8	0.54	51.1	21.6	0.85	64.8	8.2	0.32	0.44	0.17	0.007	0.15	0.25	0.010
11	59.6	11.1	0.43	41.9	19.4	0.76	71.4	14.9	0.59	49.4	19.7	0.77	64.8	9.3	0.37	0.45	0.17	0.007	0.14	0.25	0.010
7	59.2	11.2	0.44	44.0	18.8	0.74	69.3	15.1	0.59	48.9	18.6	0.73	65.0	9.4	0.37	0.46	0.16	0.006	0.14	0.24	0.010
1	57.0	11.0	0.43	50.2	20.1	0.79	61.5	15.7	0.62	46.5	14.8	0.58	64.9	10.9	0.43	0.48	0.15	0.006	0.12	0.24	0.009
13	57.8	10.9	0.43	37.4	19.1	0.75	71.4	14.5	0.57	46.6	20.4	0.80	63.1	8.9	0.35	0.41	0.17	0.007	0.09	0.25	0.010
14	57.6	10.5	0.41	37.6	18.9	0.74	70.9	14.5	0.57	46.3	19.7	0.77	63.0	8.5	0.33	0.42	0.17	0.007	0.09	0.24	0.010
12	57.0	10.5	0.41	36.5	19.1	0.75	70.6	14.0	0.55	45.3	20.2	0.79	62.5	8.5	0.33	0.40	0.17	0.007	0.07	0.25	0.010

**Table 6:** Most frequently occurring (MFO) feature subsets for turn section results and random forest classifier using 2500-iteration random-shuffle-split cross validation (2500-RSS), ordered by ranked performance. PPV: positive predictive value, NPV: negative predictive value, MCC: Matthews correlation coefficient, $\bar{x}$: mean, SD: standard deviation, CI: 95% confidence interval.

# Features	Accuracy (%)			Sensitivity (%)			Specificity (%)			PPV (%)			NPV (%)			F1			MCC		
	$\bar{x}$	SD	CI	$\bar{x}$	SD	CI	$\bar{x}$	SD	CI	$\bar{x}$	SD	CI	$\bar{x}$	SD	CI	$\bar{x}$	SD	CI	$\bar{x}$	SD	CI
5	77.3	9.1	0.36	66.1	19.6	0.77	84.7	11.4	0.45	74.3	15.5	0.61	79.0	9.7	0.38	0.70	0.15	0.006	0.52	0.20	0.008
6	77.1	9.4	0.37	66.2	19.5	0.76	84.4	11.7	0.46	73.9	15.9	0.62	78.9	9.7	0.38	0.70	0.15	0.006	0.52	0.21	0.008
3	77.0	9.6	0.38	67.7	18.9	0.74	83.2	12.2	0.48	72.9	15.7	0.62	79.5	9.7	0.38	0.70	0.14	0.006	0.52	0.21	0.008
9	76.3	9.6	0.38	63.3	19.8	0.78	84.9	11.6	0.46	73.6	16.7	0.66	77.6	9.6	0.38	0.68	0.15	0.006	0.50	0.22	0.009
2	76.4	9.4	0.37	65.9	18.9	0.74	83.4	12.0	0.47	72.6	15.8	0.62	78.6	9.6	0.38	0.69	0.14	0.006	0.50	0.20	0.008
7	75.8	9.6	0.38	62.4	19.5	0.76	84.7	12.0	0.47	73.2	16.7	0.66	77.2	9.5	0.37	0.67	0.15	0.006	0.49	0.21	0.008
8	75.7	9.7	0.38	62.4	19.7	0.77	84.5	12.0	0.47	72.9	16.9	0.66	77.1	9.6	0.38	0.67	0.15	0.006	0.48	0.22	0.009
4	75.5	9.5	0.37	63.3	19.5	0.76	83.7	11.9	0.47	72.2	16.5	0.65	77.4	9.6	0.38	0.67	0.15	0.006	0.48	0.21	0.008
1	75.3	9.4	0.37	61.5	19.5	0.76	84.6	11.7	0.46	72.7	16.8	0.66	76.7	9.3	0.36	0.67	0.15	0.006	0.48	0.21	0.008

**Table 7:** Combined straight and turn-walking feature results for 2500-iteration random-shuffle-split cross validation (2500-RSS), ordered by ranked performance. PPV: positive predictive value, NPV: negative predictive value, MCC: Matthews correlation coefficient, S5B: select-5-best method, SEL: false positive and discovery rate method, RFE: recursive feature eliminator, RF: random forest, *k*NN: *k*-nearest neighbour, SVM: support vector machine, linear: linear kernel, $\bar{x}$: mean, SD: standard deviation, CI: 95% confidence interval.

Classifier, Feature Selection	Accuracy (%)			Sensitivity (%)			Specificity (%)			PPV (%)			NPV (%)			F1			MCC		
	$\bar{x}$	SD	CI	$\bar{x}$	SD	CI	$\bar{x}$	SD	CI	$\bar{x}$	SD	CI	$\bar{x}$	SD	CI	$\bar{x}$	SD	CI	$\bar{x}$	SD	CI
RF S5B	71.6	10.8	0.42	57.5	20.8	0.82	81.1	13.2	0.52	66.9	18.9	0.74	74.1	10.2	0.40	0.62	0.17	0.007	0.40	0.25	0.010
RF SEL	69.5	11.7	0.46	54.3	21.1	0.83	79.7	14.5	0.57	64.1	20.7	0.81	72.3	10.4	0.41	0.59	0.18	0.007	0.35	0.27	0.010
*k*NN (*k* = 5) S5B	67.4	11.2	0.44	48.6	21.3	0.84	80.0	13.9	0.55	61.8	21.6	0.85	70.0	9.9	0.39	0.54	0.19	0.007	0.30	0.27	0.011
SVM (linear) S5B	65.7	11.6	0.45	56.1	21.0	0.83	72.1	15.9	0.62	57.3	17.7	0.70	71.1	11.5	0.45	0.57	0.16	0.006	0.28	0.25	0.010
*k*NN (*k* = 3) S5B	65.9	11.4	0.45	49.3	20.6	0.81	77.0	14.4	0.56	58.8	20.6	0.81	69.5	10.0	0.39	0.54	0.18	0.007	0.27	0.26	0.010
SVM (linear) SEL	63.7	12.5	0.49	54.9	23.8	0.94	69.6	17.9	0.70	54.6	19.9	0.78	69.8	13.5	0.53	0.55	0.19	0.007	0.24	0.30	0.012
*k*NN (*k* = 3) SEL	65.3	12.7	0.50	49.0	21.1	0.83	76.2	15.3	0.60	57.9	21.8	0.85	69.2	10.8	0.42	0.53	0.19	0.007	0.26	0.29	0.011
*k*NN (*k* = 5) SEL	65.4	12.5	0.49	47.2	22.0	0.86	77.5	15.1	0.59	58.3	23.3	0.91	68.8	10.7	0.42	0.52	0.20	0.008	0.26	0.29	0.012
